# Descriptive analysis of herpes zoster in childhood-onset systemic lupus erythematosus

**DOI:** 10.1186/1546-0096-10-S1-A25

**Published:** 2012-07-13

**Authors:** Josephine Isgro, Deborah M Levy, Philip LaRussa, Lisa F Imundo, Andrew H Eichenfield

**Affiliations:** 1 Morgan Stanley Children's Hospital of New York Presbyterian, Columbia University Medical Center, New York, NY, USA; 2 The Hospital for Sick Children, Toronto, ON, Canada

## Purpose

Herpes zoster is reported to be one of the most common viral infections affecting Systemic Lupus Erythematosus (SLE) patients. The aim of the present study is to provide a descriptive analysis of the clinical features seen in childhood-onset SLE (cSLE) patients affected by herpes zoster.

## Methods

A retrospective chart review of cSLE patients at one tertiary care center was conducted. Among cSLE patients followed between 1998 and January 2010, 9 patients who were diagnosed and treated for herpes zoster were identified. Data extracted included demographic and clinical features of cSLE disease at presentation, disease activity, medication and immunization history, and course of zoster infection.

## Results

A total of 170 patients with childhood-onset SLE were treated in the defined time period. 9 (5.3%) of cSLE patients developed one or more herpes zoster virus (HZV) infections. There were 8 females (89%), and the mean age of cSLE at diagnosis of HZV was 12.75 yr (± 3.18). There were 4 Hispanic patients, 3 African-American, 1 Asian, and 1 Caucasian. Distribution of skin lesions included the chest in 2 patients (T4 and T6 dermatomes, respectively), abdomen in 2, and 2 each in the upper and lower extremities. All patients had resolution of lesions within 4 weeks and there was no mortality. Two patients had visceral involvement, 1 with meningeal spread and another with pancreatitis. Two patients experienced postherpetic neuralgia requiring narcotics. All patients were treated with antiviral medications. Four were hospitalized and received acyclovir intravenously, of whom 2 continued on valacyclovir and 1 famciclovir orally. The fourth patient developed renal insufficiency after one day of acyclovir and received no further treatment. The remaining 5 patients were treated orally, 4 with valacyclovir and 1 with famciclovir. One patient was previously immunized against varicella; 5 had documentation of primary varicella disease in the past. Mean cSLE disease duration at time of HZV infection was 4.11 yr (± 2.99), and patients were receiving a mean dose of prednisone 0.6 mg/kg/d (± 0.51) at the time of infection. Prednisone dose at the time of HZV differed significantly from the preceding 3 months (p=0.01, Wilcoxon test). All patients were receiving additional immunosuppressive therapy: 4 intravenous cyclophosphamide, 2 mycophenolate mofetil, and 2 azathioprine at the time of infection. SLE disease activity as measured by SLEDAI at the time of HZV and 6 months prior to infection demonstrated a significant increase prior to infection (p=0.04, Wilcoxon test). Other markers of SLE disease activity were measured, including dsDNA, C3, C4, total leucocyte count, absolute neutrophil count, absolute lymphocyte count, and platelet count in the months prior to infection and demonstrated no significant change.

## Conclusion

cSLE patients are at increased risk for herpes zoster infections, and in our population were associated with a recent increase in disease activity and increased glucocorticoid use. Depending upon the site and extent of involvement, it can be associated with significant morbidity.

## Disclosure

Josephine Isgro: None; Deborah M. Levy: None; Philip LaRussa: None; Lisa F. Imundo: None; Andrew H. Eichenfield: None.

